# Stentless Strategy by Drug-Coated Balloon Angioplasty following Directional Coronary Atherectomy for Left Main Bifurcation Lesion

**DOI:** 10.1155/2021/5529317

**Published:** 2021-03-03

**Authors:** Norihiro Kobayashi, Masahiro Yamawaki, Shinsuke Mori, Masakazu Tsutsumi, Yohsuke Honda, Kenji Makino, Shigemitsu Shirai, Masafumi Mizusawa, Yoshiaki Ito

**Affiliations:** Department of Cardiology, Saiseikai Yokohama City Eastern Hospital, Yokohama, Japan

## Abstract

**Aims:**

We aimed to evaluate the efficacy of stentless strategy by drug-coated balloon (DCB) angioplasty following directional coronary atherectomy (DCA) for left main (LM) bifurcation lesions.

**Methods:**

A total of 38 patients who underwent DCB angioplasty following DCA for LM bifurcation lesions were retrospectively enrolled. The primary endpoint was target vessel failure (TVF) at 12 months. Secondary endpoints included procedure-related major events during the hospitalization, major adverse cardiac events at 12 months, ischemia-driven target lesion revascularization (TLR) at 12 months, and bleeding complications defined as the Bleeding Academic Research Consortium criteria ≥2 at 12 months.

**Results:**

Among these 38 lesions, 31 lesions were de novo LM bifurcation lesions and 7 lesions were stent edge restenosis at the left anterior descending (LAD) ostium. The mean % plaque area (%PA) after DCA was 44.0 ± 7.4%. TVF at 12 months occurred in 1 lesion (3.2%) of de novo LM bifurcation lesion and in 3 lesions (42.9%) of stent edge restenosis at the LAD ostium. All events of TVF were ischemia-driven TLR by percutaneous coronary intervention. Among 4 TLR cases, %PA after DCA was high (55.9%) in the de novo LM bifurcation lesions; on the other hand, %PA after DCA was low (42.4%, 38.7%, and 25.7% in the 3 cases) in stent edge restenosis at the LAD ostium. No procedure-related major events were observed during hospitalization. There was no cardiac death, no myocardial infarction, no coronary artery bypass grafting, and no bleeding complications at 12 months.

**Conclusions:**

Stentless strategy by DCB angioplasty following DCA for de novo LM bifurcation lesions resulted in acceptable outcomes. On the other hand, its efficacy was limited for stent edge restenosis at the LAD ostium even after aggressive debulking by DCA.

## 1. Introduction

Clinical outcomes of percutaneous coronary intervention (PCI) have improved with the development of drug-eluting stents (DESs) and technical advancements. However, PCI for left main (LM) bifurcation lesions remains challenging due to concerns over future revascularization [1]. Previous studies reported that a simple stent strategy had better clinical outcomes compared to a complex stent strategy even using newer generation DESs for LM bifurcation lesions [2, 3]. However, the success of a single stent strategy is usually dependent on the anatomical complexity. Complex stenting is often unavoidable especially for true bifurcation lesions with a large side branch and for true bifurcation lesions with a shallow bifurcation angle due to plaque and carina shift [4]. In addition, although the risk of developing stent thrombosis has decreased with the development of DESs, stent thrombosis at LM bifurcation lesions becomes critical due to large amount of myocardium at risk. On the other hand, major bleeding events especially for patients with a high bleeding risk, also becomes a critical issue; however, the ideal dual antiplatelet therapy (DAPT) duration after stenting for LM bifurcation lesions remains unclear. This results in a clinical dilemma for physicians as they need to balance the risk and benefits between DAPT continuation to prevent stent thrombosis or a short DAPT duration to avoid major bleeding events after stenting for LM bifurcation lesions. With the advancement of the drug-coated balloon (DCB), stentless strategy is one of the potential options to overcome thrombotic and bleeding events. A previous study reported the feasibility of the stentless strategy by rotational atherectomy following DCB angioplasty for severe calcified coronary lesions [5]. Similarly, for LM bifurcation lesions, an improved novel directional coronary atherectomy (DCA) catheter (ATHEROCUT®, Nipro Corporation, Osaka, Japan) has been commercially available in Japan since 2015 and the possibility of stentless strategy using DCA followed by the DCB angioplasty has been investigated. A recent multicenter registry which was conducted in Japan (DCA/DCB registry) reported the efficacy of the DCB angioplasty following DCA for coronary bifurcation lesions [6]. We investigated the efficacy of stentless strategy by DCB angioplasty following DCA for LM bifurcation lesions.

## 2. Methods

### 2.1. Study Design and Subjects

This was a retrospective observational study conducted at Saiseikai Yokohama-city Eastern Hospital. We retrospectively identified 78 patients with stable angina pectoris who underwent PCI using DCA from our database between April 2016 and October 2019. After the exclusion of 20 patients who underwent DCA for lesions other than LM bifurcation lesions, a total of 58 patients who underwent DCA for LM bifurcation lesion were identified. Our indications of DCA for LM bifurcation lesion were as follows: (1) stable angina pectoris with LM bifurcation lesion involving the distal LM trunk, ostium of left anterior descending (LAD), or left circumflex artery (LCX), (2) reference diameter in the main branch >2.5 mm by visual estimation, and (3) intravascular ultrasound (IVUS) findings were suitable for DCA (no lipid rich plaque, no thrombus, no severe superficial calcification, and plaque location to be debulked by DCA was accurately evaluated by IVUS). The exclusion criteria were as follows: (1) unstable angina pectoris and myocardial infarction, (2) poor general condition of the patient and renal insufficiency (Cr >1.5 mg/dl), (3) severe angled lesion, and (4) angiographical severe calcified lesion. This study was approved by the institutional review board of our hospital and complied with the Declaration of Helsinki.

### 2.2. Procedure and Follow-Up

All the procedures were performed through the femoral artery using 8Fr sheath and 8Fr guiding catheter. A bolus injection of heparin (5000 U) was given after inserting the sheath and the activated coagulation time was maintained at >300 sec with an additional bolus of heparin. Lesion morphology was assessed by IVUS after crossing the lesion by a conventional guidewire. We carefully evaluated plaque distribution to be debulked and also assessed the existence of lipid rich plaque, thrombus, and superficial calcification. The ATHEROCUT® (Nipro Corporation, Osaka, Japan) was used for all the lesions and size selection was dependent on the reference diameter identified by IVUS. DCA was started with low balloon pressure (0 or 1 atm) and the IVUS evaluation was repeated after several sessions of DCA. Balloon pressure was gradually increased according to the IVUS findings and multiple cuts were repeated to obtain residual % plaque area (%PA) <60% if possible [7]. Experienced operators carefully evaluated the IVUS and angiographical findings and determined whether stentless strategy was acceptable. When stentless strategy was acceptable, a balloon angioplasty using the DCB (SeQuent Please®, Nipro Corporation, Osaka, Japan) was performed. The size of the DCB was selected according to the reference lumen diameter by IVUS and the balloon inflation time was 30 sec with nominal pressure. Provisional stent implantation was considered at the discretion of an experienced operator when the IVUS showed large residual plaque burden, huge dissection, or hematoma formation. Dual antiplatelet therapy with 100 mg/day aspirin and either 75 mg/day clopidogrel or 3.75 mg/day prasugrel was started before the procedure and continued for 3 months after the procedure. All the patients were monitored until 30 days after discharge and following that every 2 to 3 months. Follow-up coronary angiography was scheduled at 9 to 12 months after the procedure.

### 2.3. Quantitative Coronary Angiography and IVUS

Quantitative coronary angiography (QCA) analysis was performed using computer-based software (HeartII ver2.0.2.3, GADELIUS) before the procedure, after the procedure, and at follow-up by an independent physician who was blinded to patient and procedural characteristics. Optimal views of the lesions were obtained at baseline, and the same projection angle was used at follow-up. The minimal lumen diameter (MLD), reference diameter (RD), lesion length, and percent diameter stenosis (%DS) were measured. The acute gain was defined as the increase in MLD after PCI; late lumen loss was defined as the difference between the postprocedural MLD and the MLD at follow-up. Binary restenosis was defined as %DS >50% at follow-up. All IVUS procedures were performed using commercially available IVUS catheters (OptiCross™; Boston Scientific, or ViewIT®; Terumo) with automatic pull-back at a rate of 0.5 mm/s. At the narrowest cross-section area, lumen diameter, lumen area, vessel area, and %PA were analyzed. The %PA was defined as (vessel area-lumen area) × 100/vessel area. The incidence of hematoma, intimal dissection, and medial dissection was recorded. The IVUS images were analyzed using computerized planimetry software (echoPlaque; INDEC Medical Systems, Los Altos, CA, USA). All images were independently assessed by physicians who were blinded to patient and clinical data.

### 2.4. Endpoints and Definitions

The primary endpoint was target vessel failure (TVF) at 12 months. TVF was defined as a composite of cardiac death, target vessel myocardial infarction (MI), and ischemia-driven target vessel revascularization (TVR) by PCI or coronary artery bypass grafting (CABG). Secondary endpoints included procedure-related major events during hospitalization, major adverse cardiac events (MACE) at 12 months, ischemia-driven target lesion revascularization (TLR) at 12 months, and bleeding complications which were defined as the Bleeding Academic Research Consortium criteria ≥2 at 12 months [8]. MACE were defined as a composite of cardiac death, MI, and ischemia-driven TVR. Ostial lesion of the LAD located ≤5 mm from the proximal stent edge of was defined as stent edge restenosis at the LAD ostium.

### 2.5. Statistical Analysis

Data were expressed as the mean ± standard deviation for continuous variables and categorical data were shown as numbers with percentages. Continuous variables were examined using the unpaired *t*-test or Mann–Whitney *U* test. Two-sided *P* < 0.05 was considered statistically significant. All analyses were performed using SPSS software (version 19; IBM-SPSS, Chicago, IL).

## 3. Results

### 3.1. Study Participants

We performed DCA for LM bifurcation lesions for 58 patients during April 2016 to October 2019. We enrolled 38 patients who underwent stentless strategy by DCB angioplasty following DCA for LM bifurcation lesions after excluding 20 patients (18 patients: DES implantation after DCA and 2 patients: DCA alone). Among 38 lesions, 31 lesions were de novo LM bifurcation lesions and 7 lesions were stent edge restenosis at the LAD ostium.

### 3.2. Baseline Characteristics and Procedural Results

Table 1 describes the baseline characteristics. The mean patient age was 70 ± 9 years and 89% of the cohort was male. The most frequent lesion classification was Medina (0, 1, 0) (68%) followed by Medina (1, 1, 0) (15%). The main target of DCA was the LAD ostium (71%), followed by both distal LM trunk and LAD ostium (13%).  Table 2 summarizes the procedural results. Size *L* of the DCA catheter was the most frequently used (87%). The mean number of cuts was 27 ± 17 times and the maximum balloon pressure of the DCA catheter was 3.7 ± 1.3 atm. The DCB angioplasty was performed after DCA for all lesions and the diameter of the DCB was 3.3 ± 0.4 mm and balloon pressure was 8.4 ± 2.8 atm. In the QCA analysis, MLD and % DS improved significantly after the procedure (MLD: 1.3 ± 0.5 mm versus 3.4 ± 0.9 mm, *P* < 0.001; % DS: 63 ± 11% vs. 11 ± 8%, *P* < 0.001 (Table 3).  Table 4 summarizes the IVUS findings during the procedure. Lumen area increased significantly (pre-DCA: 3.1 ± 1.0 mm^2^ versus post-DCA: 8.6 ± 2.0 mm^2^, *P* < 0.001) and %PA decreased significantly after DCA (pre-DCA: 76.2 ± 7.1% versus post-DCA: 44.0 ± 7.4%, *P* < 0.001). Intimal dissection was observed in 5 lesions (13%); however, there was no medial dissection or hematoma formation. During the procedure, no complications including vessel perforation, slow flow phenomenon, and stuck of the DCA catheter occurred. There were no procedure-related major events including cardiac death, MI, any emergent revascularization, and access site problems during hospitalization (Table 5).

### 3.3. Follow-Up Results

Angiographic follow-up was performed for 35 patients (angiographic follow-up rate: 92.1%). At the follow-up coronary angiography, MLD and % DS were similar to that after the procedure (MLD: 3.2 ± 1.1 mm versus 3.4 ± 0.9 mm, *P*=0.48; % DS: 17 ± 15% versus 11 ± 8%, *P*=0.30) (Table 3). Table 5 summarizes the clinical follow-up results. TVF at 12 months occurred in 4 patients (10.5%) and the cause of TVF across all 4 cases was ischemia-driven TVR by PCI. In addition, TVR resulted from ischemia-driven TLR. For de novo LM bifurcation lesions, the incidence of binary restenosis and ischemia-driven TLR was 6.3% (2 lesions/32 lesions) and 3.2% (1 lesion/32 lesions), respectively. On the other hand, for stent edge restenosis at the LAD ostium, the incidence of binary restenosis and ischemia-driven TLR was 42.9% (3 lesions/7 lesions) and 42.9% (3 lesions/7 lesions), respectively. No MACE, except for ischemia-driven TVR, were observed at 12 months. In addition, there were no bleeding complications at 12 months.

### 3.4. IVUS Findings and Ischemia-Driven TLR at 12 Months

**Figure 1:**
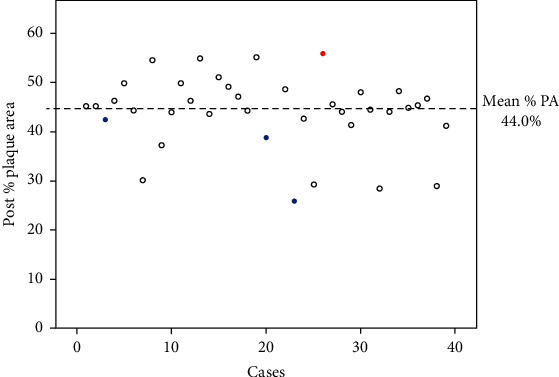
Distribution of % PA after DCA for each case. Mean %PA in the overall population was 44.0%. The red dot shows ischemia-driven TLR case at 12 months for the de novo LM bifurcation lesion after DCA. The blue dots show ischemia-driven TLR cases at 12 months for stent edge restenosis at the LAD ostium after DCA.

**Figure 2:**
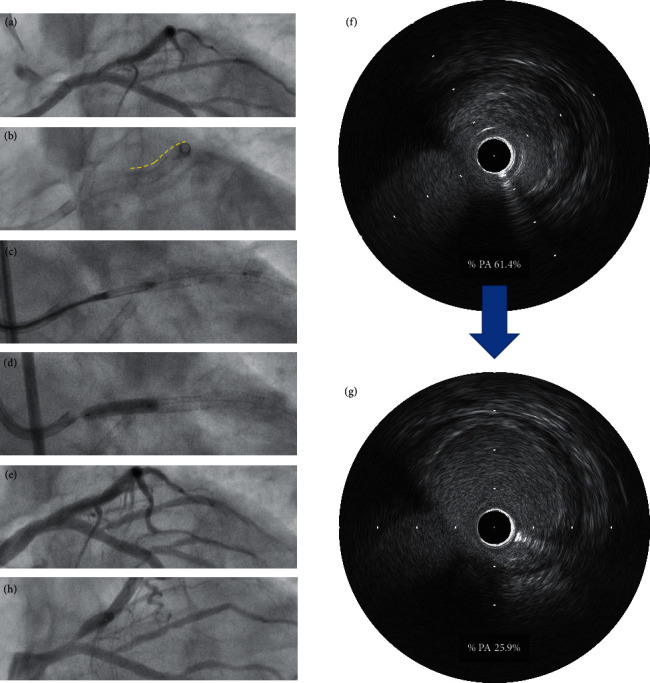
Representative case of ischemia-driven TLR after DCA for stent edge restenosis at the LAD ostium: (a) preprocedure coronary angiography, (b) yellow dotted line indicates previously implanted stent, (c) DCA was performed for stent proximal edge restenosis at the LAD ostium, (d) DCB angioplasty was performed, (e) postprocedure coronary angiography, (f) IVUS findings at preprocedure shows %PA was 61.4%, (g) IVUS findings after DCA shows %PA decreased to 25.9%, and (h) follow-up coronary angiography at 10 months.

Figure 1 shows the distribution of %PA for each case. Ischemia-driven TLR was performed for 4 lesions; for 1 de novo LM bifurcation lesion (red dot) and for 3 stent edge restenosis at the LAD ostium (blue dots). The %PA of the de novo LM bifurcation lesion with TLR (55.9%) was larger compared to the mean %PA (44.0%). On the other hand, the %PA of the stent edge restenosis at the LAD ostium with TLR (42.4%, 38.7%, and 25.7%) was lower than mean %PA.  Figure 2 shows a representative case of TLR after DCA for stent edge restenosis at the LAD ostium. Preprocedure coronary angiography showed stent edge restenosis at the LAD ostium (Figures 2(a) and 2(b)). A DCA with size *L* was performed and DCB angioplasty using 3.5 mm diameter by 15 mm was followed (Figures 2(c) and 2(d)). Postprocedure coronary angiography showed improved stenosis (Figure 2(e)). The %PA identified by IVUS decreased to 25.9% from 61.4% (Figures 2(f) and 2(g)). However, the follow-up angiography at 10 months revealed restenosis at the LAD ostium (Figure 2(h)).

## 4. Discussion

The main findings of the current study were as follows. (1) The mean %PA after DCA for LM bifurcation lesions was 44.0% and the incidence of TVF at 12 months was low (3.1%) for de novo LM bifurcation lesions. On the other hand, the incidence of TVF was high (42.9%) for stent edge restenosis at the LAD ostium. All TVF resulted from ischemia-driven TLR. (2) There were no procedure-related major events during hospitalization, no cardiac death, no MI, and no bleeding events at 12 months. (3) The %PA of de novo LM bifurcation with TLR was higher (55.9%) compared to the mean %PA. On the other hand, for stent edge restenosis at the LAD ostium, TLR was required even with low %PA after DCA (42.4%, 38.7%, and 25.7%).

The ABACAS study compared the incidence of TLR between balloon angioplasty after aggressive DCA and DCA alone, and there was no difference between the groups [9]. The %PA after DCA of the ABACAS study was 45.6% and this was almost similar to that of the current study (44.0%). However, our incidence of TLR at 12 months was extremely low compared to the ABACAS study (3.2% versus 20.6%). We consider that DCB angioplasty played an important role in inhibiting neointimal hyperplasia. On the other hand, a recent multicenter registry (DCA/DCB registry) demonstrated the low incidence of TLR at 12 months (3.6%) after DCA following DCB angioplasty for coronary bifurcation lesions even though the mean %PA after DCA was higher than our study (56.3% versus 44.0%) [6]. Based on these results, aggressive debulking of DCA to achieve a %PA less than 50% may not be necessary, if DCA was followed by DCB angioplasty. Although the definition of an optimal target %PA after DCA is difficult, we reckon that the optimal target %PA may be 50 to 55% for de novo LM bifurcation lesions as we experienced one TLR case in which the %PA after DCA was 55.9%. However, 55.9% of %PA is similar to the mean %PA of the DCA/DCB registry; therefore, not only %PA after DCA but also other factors such as severity of residual dissection, lumen area, and plaque morphology may be associated with restenosis after stentless strategy for LM bifurcation lesions. Further investigations are needed to clarify this issue.

Stent thrombosis at LM bifurcation lesions is critical; therefore, it is important to decrease the risk of stent thrombosis as low as possible. In general, the incidence of stent thrombosis has decreased with the development of DES technology. However, it is difficult to nullify its incidence. Furthermore, optimal DAPT duration to avoid stent thrombosis and major bleeding events after stenting for LM bifurcation lesions remains unclear. We reckon that stentless strategy using DCB angioplasty following DCA is an acceptable option to resolve these issues. In particular, there were no procedure-related major events during hospitalization, no MACE except for ischemia-driven TLR at 12 months, and no bleeding events at 12 months in the current study. In addition, although we stopped DAPT at 3 months after the procedure, optimal DAPT duration after DCB angioplasty may shorten according to the future clinical studies. The DCA procedure requires a skilled operator. However, if stentless strategy for LM bifurcation lesions succeeds, it may be beneficial compared to stent implantation for LM bifurcation lesion.

The current study demonstrated acceptable results of DCB angioplasty following DCA for de novo LM bifurcation lesions. However, the results of this strategy for stent edge restenosis at the LAD ostium were poor. Currently, data in relation to the efficacy of DCB angioplasty following DCA for stent edge restenosis is lacking. A previous study reported that there was a tendency towards higher TLR following DCB angioplasty compared to repeat implantations of DESs for stent edge restenosis [10]. We reckon that sufficient plaque debulking and DCB angioplasty are effective to obtain large lumen area and to inhibit neointimal hyperplasia which is the main cause of restenosis in nonstented vessels. On the other hand, several mechanisms of stent edge restenosis have been reported including negative remodeling, mechanical injury at stent edge due to hinge motion, and local changes in shear stress [11–13]. In particular, all the TLR cases in the current study of stent edge restenosis at the LAD ostium had a low %PA following DCA. Therefore, these factors may contribute to restenosis. To overcome these factors, repeat stent implantation may be a favorable strategy for stent edge restenosis at the LAD ostium. However, stentless strategy without LM stenting is advantageous in terms of shortening DAPT duration and lowering the risk of stent thrombosis. The current study failed to demonstrate the efficacy of DCB angioplasty following DCA for stent edge restenosis at the LAD ostium. However, only a small number of patients were analyzed. Further research with a large sample number is required to confirm the results of the present study.

### 4.1. Study Limitations

This study has several limitations. First, this was a retrospective analysis of a single center and sample volume, especially the number of patients with stent edge restenosis at the LAD ostium was small. Selection bias should be considered and other large-scale studies are needed to clarify the clinical impact of stentless strategy for LM bifurcation lesions. Second, we performed only IVUS after DCA to evaluate the lumen area and dissection formation. As such, we were unable to categorize the dissection severity. It is well known that optical coherence tomography (OCT) is superior to IVUS to evaluate detailed dissection morphology [14]. Therefore, further evaluations using OCT to investigate the characteristics of dissection are required. However, we reckon that DCA should be performed under the guidance of IVUS and not OCT because OCT is unable to provide information about vessel size and plaque volume, thereby limiting its safety. Third, the DCA procedure requires a specific technique in IVUS interpretation and manipulation of the DCA catheter, thereby limiting the applicability and generalizability of the results from the current study. Finally, our follow-up data was limited to 12 months. To evaluate the efficacy of stentless strategy of DCB angioplasty following DCA for LM bifurcation lesions, future trials with a longer follow-up are necessary.

## 5. Conclusions

The early results of stentless strategy by DCB angioplasty following DCA for de novo LM bifurcation lesions are acceptable. On the other hand, this stentless strategy may not be effective for stent edge restenosis at the LAD ostium.

## Figures and Tables

**Table 1:** Baseline characteristics.

Patient characteristics	*N* = 38
Age (years)	70 ± 9
Male (%)	34 (89)
Hypertension (%)	28 (74)
Diabetes mellitus (%)	10 (26)
Hyperlipidemia (%)	31 (82)
Hemodialysis (%)	1 (3)
Current smoking (%)	2 (5)
Previous PCI (%)	17 (45)
Previous CABG (%)	0 (0)

Medication	
ACE/ARB (%)	24 (63)
*β*-Blocker (%)	25 (66)
Statin (%)	37 (97)
Aspirin (%)	38 (100)
Clopidogrel (%)	14 (37)
Prasugrel (%)	16 (42)

Medina classification	
(0, 1, 0) (%)	26 (68)
(0, 0, 1) (%)	3 (8)
(1, 0, 0) (%)	1 (3)
(1, 1, 0) (%)	6 (15)
(1, 0, 1) (%)	1 (3)
(1, 1, 1) (%)	1 (3)

Main target of DCA	
LAD ostium (%)	27 (71)
LCX ostium (%)	3 (8)
Distal LM trunk (%)	2 (5)
Distal LM trunk and LAD ostium (%)	5 (13)
Distal LM trunk, LAD ostium, and LCX ostium (%)	1 (3)

**Table 2:** Procedural results.

DCA	*N* = 38
Size	
*M* (%)	5 (13)
*L* (%)	33 (87)
Total number of cuts (times)	27 ± 17
Maximum number of cuts (times)	78
Max balloon pressure (atm)	3.7 ± 1.3

DCB angioplasty	
Diameter (mm)	3.3 ± 0.4
Length (mm)	17.6 ± 3.2
Balloon pressure (atm)	8.4 ± 2.8
Procedure time (min)	124 ± 39
Amount of contrast media (ml)	194 ± 71

Complications	
Perforation (%)	0 (0)
Slow flow phenomenon (%)	0 (0)
Stuck of the DCA catheter (%)	0 (0)

**Table 3:** Quantitative coronary analysis.

Preprocedure	*N* = 38
Minimum lumen diameter (mm)	1.3 ± 0.5
Reference lumen diameter (mm)	3.8 ± 1.1
% diameter stenosis (%)	63 ± 11
Lesion length (mm)	17.3 ± 7.2

Post procedure	
Minimum lumen diameter (mm)	3.4 ± 0.9
Acute gain (mm)	2.0 ± 1.0
Reference lumen diameter (mm)	3.8 ± 1.0
% diameter stenosis (%)	11 ± 8

Follow-up	
Minimum lumen diameter (mm)	3.2 ± 1.1
Late loss (mm)	0.2 ± 0.5
% diameter stenosis (%)	17 ± 15

**Table 4:** Intravascular ultrasound findings.

Pre-DCA	*N* = 38
Minimum lumen diameter (mm)	1.7 ± 0.3
Lumen area (mm^2^)	3.1 ± 1.0
Vessel area (mm^2^)	13.2 ± 3.5
% plaque area (%)	76.2 ± 7.1

Post-DCA	
Minimum lumen diameter (mm)	2.8 ± 0.4
Lumen area (mm^2^)	8.6 ± 2.0
Vessel area (mm^2^)	15.5 ± 3.8
% plaque area (%)	44.0 ± 7.4
Intimal dissection (%)	5 (13)
Medial dissection (%)	0 (0)
Hematoma (%)	0 (0)

**Table 5:** Clinical outcomes.

	Overall *N* = 38	De novo LM bifurcation lesion *N* = 31	Stent edge restenosis at LAD ostium *N* = 7
Procedure-related major events during the hospitalization			
Cardiac death (%)	0 (0)	0 (0)	0 (0)
MI (%)	0 (0)	0 (0)	0 (0)
Any emergent revascularization (%)	0 (0)	0 (0)	0 (0)
Access site problems (%)	0 (0)	0 (0)	0 (0)

Clinical outcomes at 12 months			
TVF at 12 months (%)	4 (10.5)	1 (3.2)	3 (42.9)
Cardiac death (%)	0 (0)	0 (0)	0 (0)
Target vessel MI (%)	0 (0)	0 (0)	0 (0)
Ischemia-driven TVR by PCI or CABG (%)	4 (10.5)	1 (3.2)	3 (42.9)
MACE at 12 months (%)	4 (10.5)	1 (3.2)	3 (42.9)
Cardiac death (%)	0 (0)	0 (0)	0 (0)
MI (%)	0 (0)	0 (0)	0 (0)
Ischemia-driven TVR (%)	4 (10.5)	1 (3.2)	3 (42.9)
Ischemia-driven TLR at 12 months (%)	4 (10.5)	1 (3.2)	3 (42.9)
Bleeding complications at 12 months (%)	0 (0)	0 (0)	0 (0)

## Data Availability

The data used to support the findings of this study are restricted by the Ethics Committee of Saiseikai Yokohama City Eastern Hospital in order to protect patient privacy and are available from the corresponding author upon request.
